# Neutrophil extracellular traps promote scar formation in post-epidural fibrosis

**DOI:** 10.1038/s41536-020-00103-1

**Published:** 2020-10-30

**Authors:** Zhen Jin, Jinpeng Sun, Zeyuan Song, Kun Chen, Yap San Min Nicolas, Rupesh KC, Qiyun Ma, Jun Liu, Mingshun Zhang

**Affiliations:** 1 Department of Orthopaedics, The Second Affiliated Hospital of Nanjing Medical University, Nanjing, Jiangsu 210011 China; 2 Department of Respiratory and Critical Care Medicine, The First Affiliated Hospital of Nanjing Medical University, Nanjing, 210029 China; 3 NHC Key Laboratory of Antibody Technique, Department of Immunology, Nanjing Medical University, Nanjing, 211166 China

**Keywords:** Neuropathic pain, Immune cell death

## Abstract

Low back pain following spine surgery is a major complication due to excessive epidural fibrosis, which compresses the lumbar nerve. The mechanisms of epidural fibrosis remain largely elusive. In the drainage samples from patients after spine operation, neutrophil extracellular traps (NETs) and NETs inducer high-mobility group box 1 were significantly increased. In a mouse model of laminectomy, NETs developed in the wound area post epidural operation, accompanied with macrophage infiltration. In vitro, macrophages ingested NETs and thereby increased the elastase from NETs via the receptor for advanced glycation end product. Moreover, NETs boosted the expression of fibronectin in macrophages, which was dependent on elastase and could be partially blocked by DNase. NF-κB p65 and Smad pathways contributed to the increased expression fibronectin in NETs-treated macrophages. In the mouse spine operation model, post-epidural fibrosis was significantly mitigated with the administration of DNase I, which degraded DNA and cleaved NETs. Our study shed light on the roles and mechanisms of NETs in the scar formation post spine operation.

## Introduction

Excessive scar formation is one of the main concerns in spine surgery. Scar tissue in the injured areas stabilizes the adjacent tissues. Post-epidural fibrosis and the consequent firm adhesions to dura mater, however, may cause low back pain^1^ and even hinder secondary surgeries. Numerous biological methods to reduce scar formation after spine surgery have been postulated by our team^2–4^ and other labs^5–7^. The mechanisms behind post-epidural fibrosis remain largely elusive.

As the most abundant leukocytes in the blood, neutrophils migrate into injured tissues and contribute to wound healing^8^. Neutrophil extracellular traps (NETs), extracellular fibers composed of DNA, and granular enzymes, were once considered a weapon of neutrophils for trapping and killing microbes^9^. In addition to pathogen components, endogenous danger-associated molecular patterns, including high-mobility group box 1 (HMGB1) from injured tissues^10^ or cancer cells^11^, may also trigger the production of NETs. Ongoing efforts have expanded the roles of NETs into diverse diseases, including autoimmune diseases^12^, pulmonary diseases^13^, and cancer metastasis^14^. Moreover, NETs have been linked to cystic fibrosis^15^, but may impair wound healing in diabetes^16^. NETs and associated enzymes promote the differentiation of fibroblasts into myofibroblasts accompanied with increased α smooth muscle actin (α-SMA) and collagen I (ref. ^17^). In contrast, NETs cleaved extracellular matrix and degraded fibronectin in the developing lung and epithelial cells^18^. The double-edged roles of NETs in post-epidural fibrosis have not been well documented.

Macrophages infiltrating into wounds are plastic in tissue repair^19^. In response to local stimuli, macrophages secreting different effector cytokines transit from pro-inflammatory in early phase to pro-reparative in late phase of wound healing^20^. In addition, macrophages may trans-differentiate into other cells, such as the macrophage to myofibroblast transition (MMT) in renal fibrosis^21^. As professional phagocytes, macrophages may actively but silently engulf and degrade NETs^22,23^. Excessive formation of NETs, however, may prime macrophages for inflammatory cytokine production, contributing to atherosclerosis^24^, and acute respiratory distress syndrome (ARDS)^25^. In the present study, we asked whether NETs were elevated in post epidural operation scar tissues and examined the roles of NETs in the fibrosis in a mouse model of laminectomy. We also sought to determine the therapeutic roles of DNase I in a mouse mode of spine operation.

## Results

### Elevated HMGB1 and NETs in clinical samples post spine surgery

**Fig. 1:**
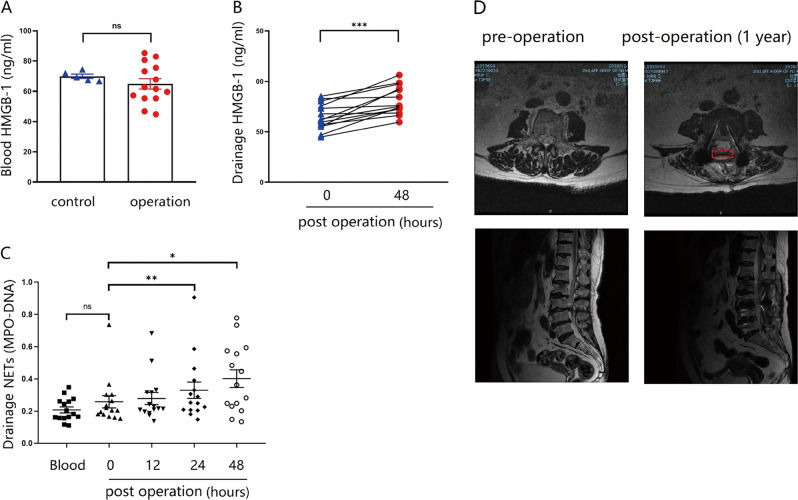
Elevated HMGB1 and NETs in clinical samples from spine operations. **A** The level of HMGB1 in the blood was similar between healthy controls and patients after spine operation; **B** Forty-eight hours after the spine operation, HMGB1 was elevated in the drainage fluid; **C** blood samples were taken immediately after the operation, and drainage samples were collected at various time points. NETs in the drainage were significantly increased after spine operation. **D** The patient experienced low back pain 1 year after the lumbar laminectomy. Retrospective analysis showed NETs were increased in the postoperative drainage. MRI results of the lumbar spine before and 1 year after the operation evidenced hyperplasia of scar tissue in the epidural after laminectomy, indicated with red dashed box. Error bars = SEM; Student’s *t* test for two groups comparison, two-way ANOVA for three and more groups comparison, number indicated in the figure; ns not significant; *p < 0.05; **p < 0.01; ***p < 0.001.

To explore whether NETs were involved with post-epidural fibrosis, we sampled peripheral blood and wound drainage from patients receiving spine surgery. HMGB1 in the plasma was comparable between the healthy controls and the patients (Fig. 1a), suggesting that spine operation caused negligible systemic inflammation. HMGB1 in the wound drainage, however, was significantly elevated 48 h after the spine operation (Fig. 1b). Moreover, NETs gradually increased in the early phase post spine operation (Fig. 1c). In the follow-up study, we noticed a patient with high NETs developed excessive epidural fibrosis (Fig. 1d), suggesting that NETs, may influence the process of scar formation in the patients receiving spine operation.

### NETs developed in the dura area in a mouse model of post laminectomy

**Fig. 2:**
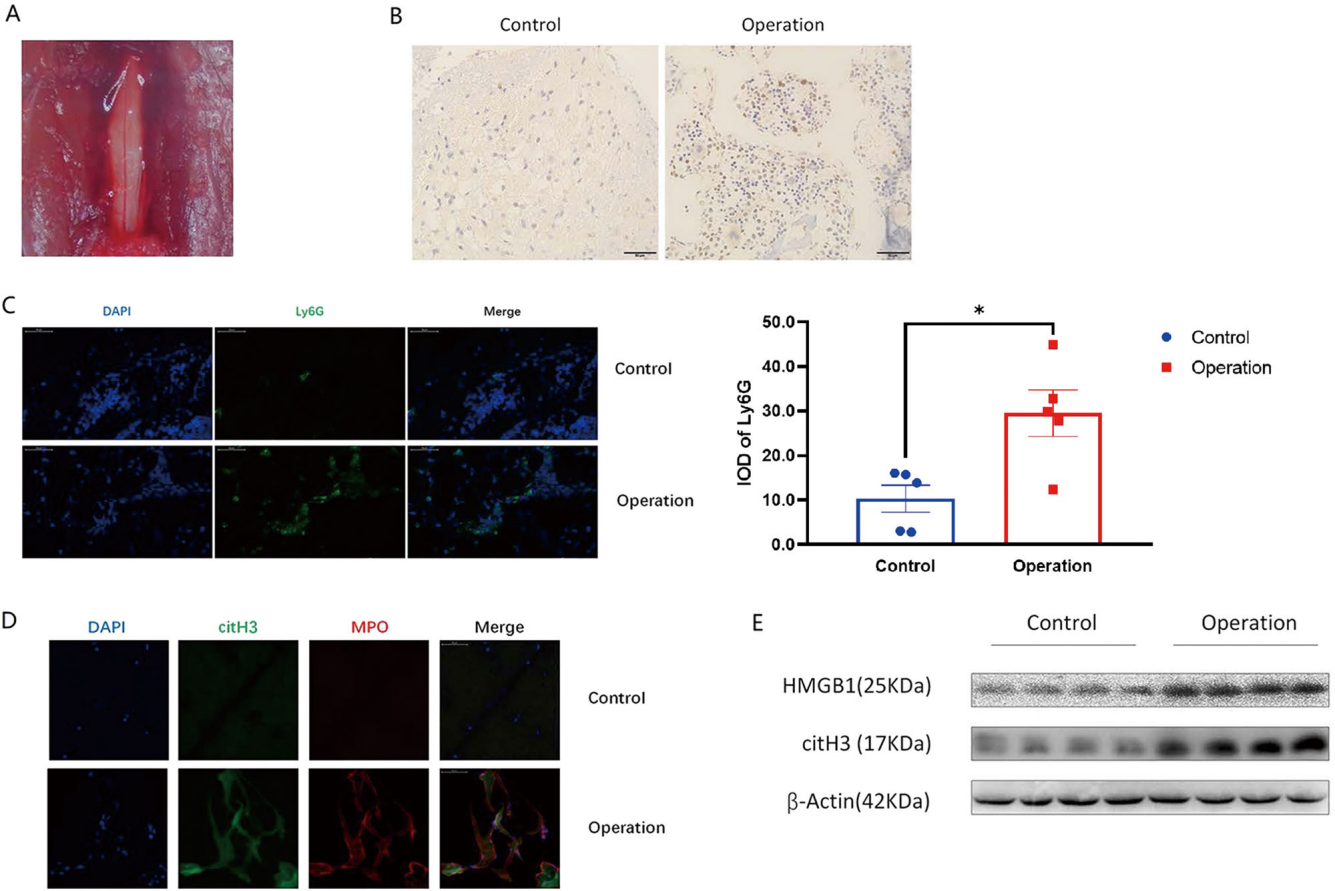
Neutrophil infiltration and NETs formation in the mouse laminectomy model. **A** After the removal of L1–L3, the dura was exposed; **B**, **C** 2 days post operation, immunohistochemistry and immunofluorescence staining revealed Ly6G-positive neutrophils accumulated in the dura area in the operation group; **D** NETs overlapped with citH3 (pseudo-red) and MPO (pseudo-green) staining in the dura area in the operation group; **E** HMGB1 and citH3 were elevated in the surgical tissues from the operation group. All blots or gels derive from the same experiment and that they were processed in parallel. Error bars = SEM; Student’s *t* test, number indicated in the figure; *p < 0.05.

After the spine operation, the dura was exposed (Fig. 2a), and the bleeding was staunched with sterile cotton. Two days after the spine operation, the tissues attached to the exposed dura were sampled. As expected, neutrophils, which are first responders in acute inflammation, were recruited into the operation area (Fig. 2b, c). Furthermore, fibrous structures co-stained with citH3 and myeloperoxidase (MPO) were identified in the operation group (Fig. 2d), suggesting that NETs developed in the wound tissues after the spine operation. As a common marker of inflammation^26^, HMGB1 is a potential inducer of NETs^10^. Accordingly, HMGB1 was elevated in the tissues attached to the exposed dura in the operation group, accompanied by increased NET marker protein citH3 (Fig. 2e). Collectively, neutrophils infiltrated into wound tissues and NETs developed in the mouse laminectomy model.

### Macrophages ingested NETs via RAGE

**Fig. 3:**
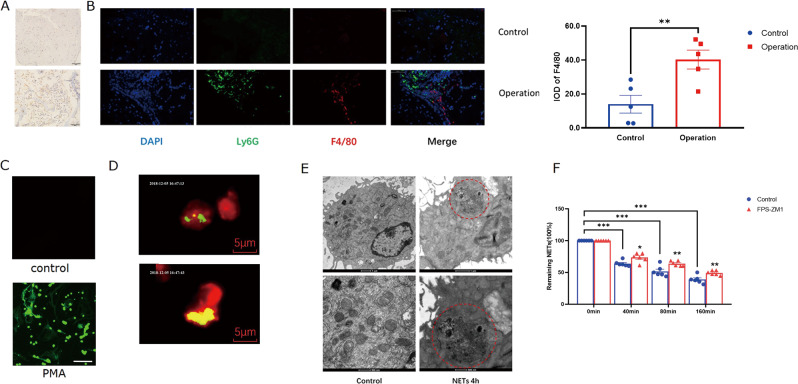
Macrophages engulfed NETs. **A** Two days post surgery, immunohistochemistry analysis revealed that F4/80-positive macrophages infiltrated the dura area in the operation group; **B** both neutrophils (Ly6G, green) and macrophages (F4/80, red) were found in the wounds of the operation group, scale bar = 50 µm; **p < 0.01; **C** mouse neutrophils were stimulated with PMA 4 h and then were stained with SYTOX Green. Original 400× magnification; scale bar = 50 µm. **D** NETs were labeled with Sytox green (green); macrophages were labeled with DDAO-SE (red); live cell imaging showed that NETs became yellow if ingested by macrophages; **E** transmission electron microscopy revealed that NETs were observed in the cytoplasm of macrophages; **F** the clearance of NETs by macrophages was reduced with the RAGE inhibitor FPS-ZM1. Error bars = SEM; Student’s *t* test for two groups comparison, two-way ANOVA for three and more groups comparison, number indicated in the figure; *p < 0.05; **p < 0.01; ***p < 0.001.

As one of the essential players in wound repair^27^, macrophages were recruited into the wound tissues 2 days post spine operation (Fig. 3a). Notably, some macrophages and neutrophils in the wound tissues were adjacent (Fig. 3b), raising the possibility that macrophages and neutrophils may be involved in epidural fibrosis. Indeed, macrophages ingested the NETs, which was directly observed by live cell imaging (Fig. 3c and Supplementary video 1) and further confirmed by transmission electron microscopy (Fig. 3d). As reported previously, endothelial cells may uptake NETs via receptor for advanced glycation end product (RAGE)^28^, and a RAGE inhibitor suppressed the ingestion of NETs by macrophages (Fig. 3e), suggesting that NETs clearance by macrophages was RAGE dependent. In summary, macrophages infiltrated into the wound area and ingested NETs after the spine operation.

### NETs increased the expression of α-SMA and fibronectin in macrophages

**Fig. 4:**
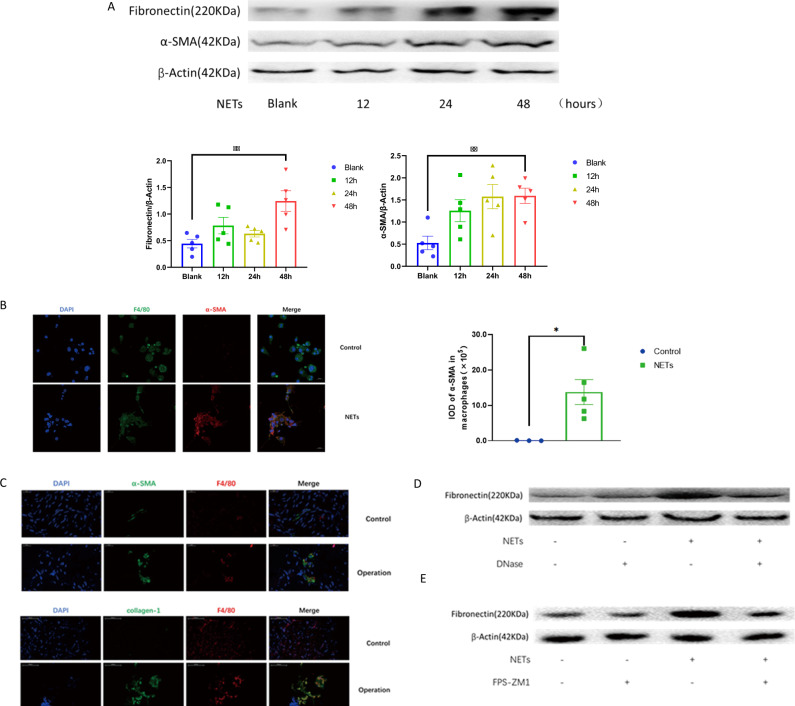
NETs promoted the macrophage phenotype transition. **A** NETs increased the expression of α-SMA and fibronectin in macrophages. **B** In vitro immunofluorescence analysis revealed that NETs-treated macrophages coexpressed F4/80 and α-SMA. **C** In the spinal tissues 2 days post operation, cells co-expressing F4/80 and α-SMA were observed. Similarly, cells coexpressed F4/80 and collagen I were also recorded. **D** DNase I reduced the expression of fibronectin in NETs-treated macrophages. **E** The RAGE inhibitor FPS-ZM1 reduced fibronectin expression in NETs-treated macrophages. All blots or gels derive from the same experiment and that they were processed in parallel. Error bars = SEM; Student’s *t* test for two groups comparison, two-way ANOVA for three and more groups comparison, number indicated in the figure; *p < 0.05; **p < 0.01.

In the process of wound healing, the extracellular matrix components α-SMA and fibronectin are released from various cells. In endothelial cells, NETs drive the mesenchymal transition^28^. As α-SMA and fibronectin are increased in the mesenchymal transition^29^, NETs enhanced the expression of α-SMA and fibronectin in macrophages (Fig. 4a). As expected, cells co-expressing the macrophage marker F4/80 and the myofibroblast marker α-SMA were identified in NETs-treated macrophages (Fig. 4b). In the wound tissues from the spine operation (Fig. 4c), cells co-stained with F4/80 and α-SMA or collagen I were accordingly increased. DNase I, which broke the DNA backbone of the NETs, reduced the effects of the NETs on the upregulation of fibronectin in macrophages (Fig. 4d), further suggesting that the elevation of fibronectin was dependent on NETs. Similarly, the RAGE inhibitor FPS-ZM1, which reduced the ingestion of NETs by macrophages, partially blocked the effect of NETs on the elevation of fibronectin (Fig. 4e). Collectively, NETs increased the expression of α-SMA and fibronectin in macrophages.

### Elevation of fibronectin was dependent on elastase in NETs and NF-κB/Smad3 pathways

**Fig. 5:**
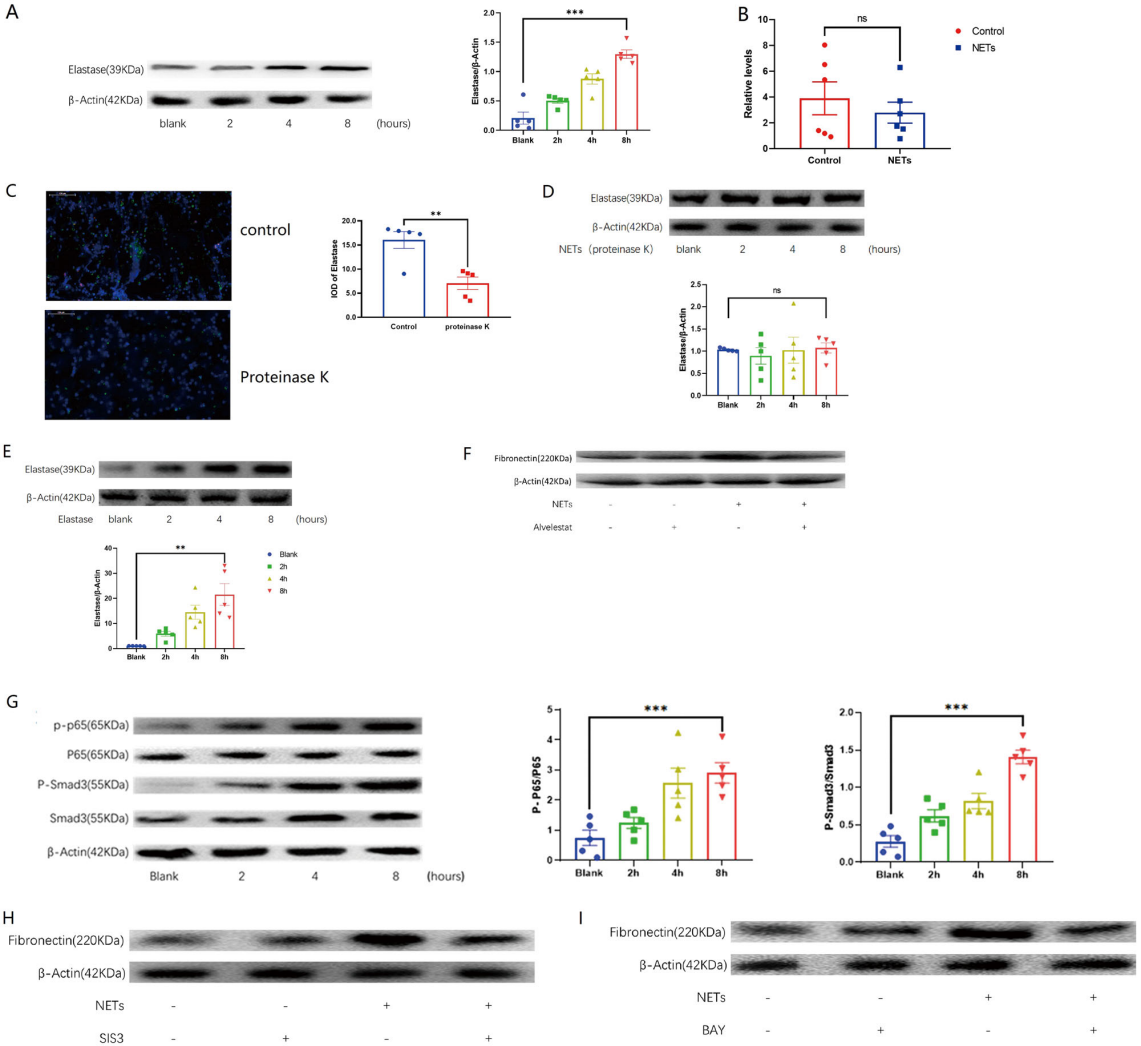
Elastase and signaling pathways involved in the macrophage phenotype transition. **A** After NETs and macrophages are cocultured, the elastase in macrophages will increase; **B** the elastase RNA level of macrophages cocultured with NETs did not change significantly; **C** proteinase K can reduce elastase in NETs; **D** after NETs were treated with proteinase K and cocultured with macrophages, the elastase of macrophages did not increase significantly; **E** after elastase was cocultured with macrophages, the elastase in macrophages was also increased; **F** alvelestat (elastase inhibitor) decreased the expression of elastase in the NETs-treated macrophages; **G** NETs activated Smad3 and P65 in macrophages; **H**, **I** SIS3 (Smad3 inhibitor) or BAY-11-7082 (P65 inhibitor) mitigated the effects of NETs on the expression of fibronectin in macrophages. All blots or gels derive from the same experiment and that they were processed in parallel. Error bars = SEM; Student’s *t* test for two groups comparison, two-way ANOVA for three and more groups comparison, number indicated in the figure; ns not significant; *p < 0.05; **p < 0.01; ***p < 0.001.

Elastase is one of the proteins decorating the NETs^30^. Interestingly, elastase promotes fibrosis in lung disease^31^. Elastase inhibition decreases scar formation after spinal cord injury^32^. In line with the above observations, NETs increased the protein level of elastase in macrophages (Fig. 5a). Elastase transcripts level was comparable in the macrophages treated with or without NETs (Fig. 5b), suggesting that the elevation of elastase protein may be derived from NETs. Indeed, proteinase K degraded elastase in the NETs (Fig. 5c) and abolished the elevation of elastase in the NETs-treated macrophages (Fig. 5d). In contrast, exogenous elastase alone was sufficient to cause the elevation of elastase in the macrophages (Fig. 5e). Further, the elastase inhibitor alvelestat blocked the increase in fibronectin in NETs-treated macrophages (Fig. 5f), suggesting that elastase from NETs may be essential in the function shift of macrophages. NF-κB^33^ and Smad3 (ref. ^6^) were two important signaling molecules contributing to epidural fibrosis. As expected, NF-κB p65 and Smad3 were activated in NETs-treated macrophages (Fig. 5g). Expectedly, the p65 inhibitor BAY-11-7082 or Smad3 inhibitor SIS3 reduced fibronectin in macrophages stimulated with NETs (Fig. 5h, i). Overall, fibronectin expression in macrophages treated with NETs was dependent on elastase and the NF-κB/Smad3 signaling pathways.

### DNase I reduced epidural fibrosis in the mouse model of laminectomy

**Fig. 6:**
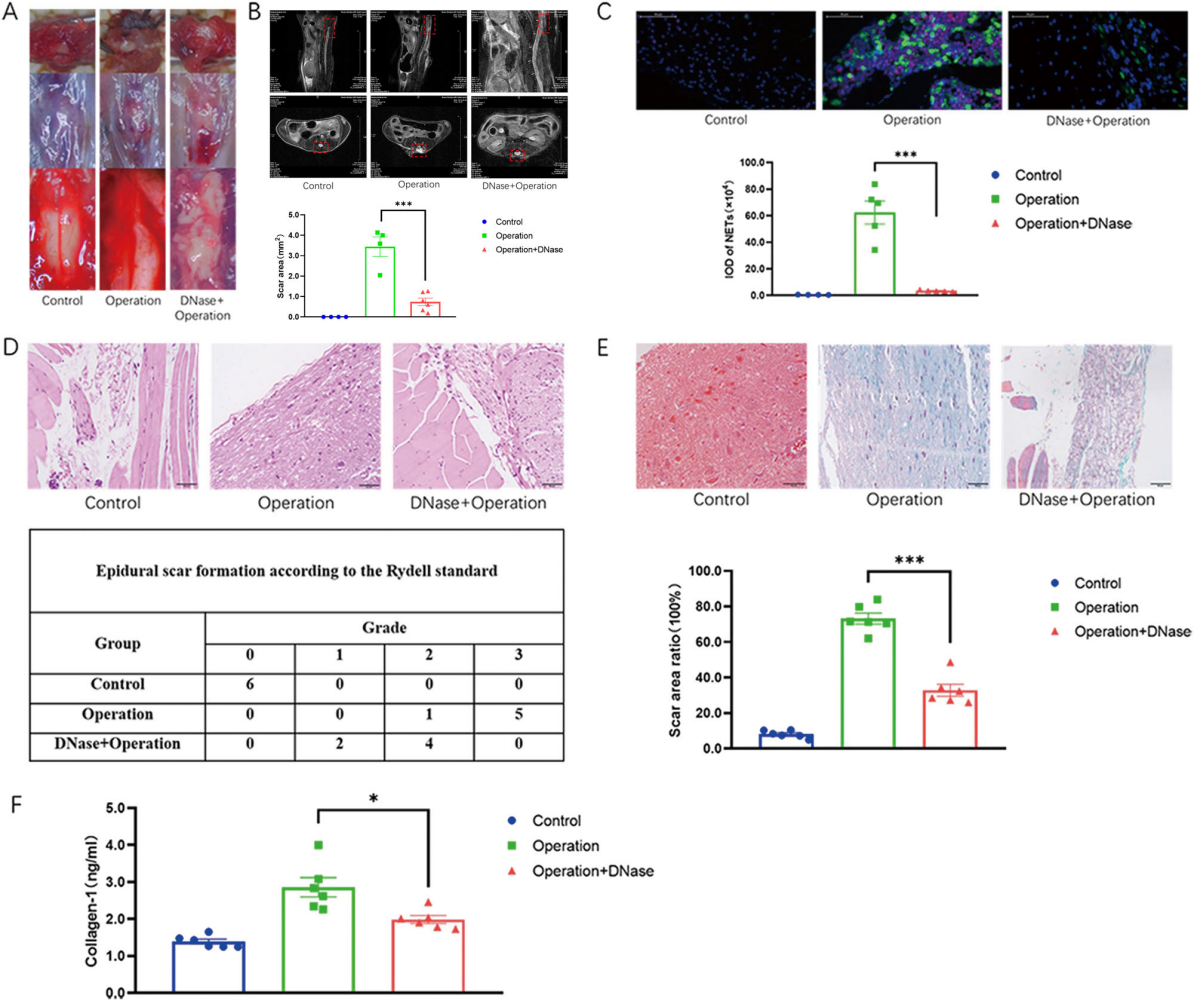
DNase I alleviated epidural fibrosis in the mouse model of laminectomy. **A** Gross observation demonstrated that DNase I reduced scar formation in the operation group; **B** MRI confirmed that there was a gap between the scar tissue and the dura mater after DNase treatment, indicating that the degree of scar adhesion decreased; **C** immunofluorescence staining attested that DNase reduced the generation of NETs (citH3, pseudo-red; MPO, pseudo-green) in the wound area after spine operation; **D** H&E staining showed the histological morphology of each group of scars. The values within the table represented the number of mice. **E** Masson staining showed the accumulation of collagen fibers in each group of scars; **F** DNase I reduced collagen I in the scar tissue from the operation group. Error bars = SEM; Student’s *t* test for two groups comparison, two-way ANOVA for three and more groups comparison, number indicated in the figure; *p < 0.05; ***p < 0.001.

In various disease models, DNase effectively degraded NETs and antagonized the effects of NETs^34,35^. A mouse model of laminectomy was established, and gross observation revealed that the scar formation was reduced in the mice treated with DNase I 30 days post operation (Fig. 6a). Magnetic resonance imaging (MRI) analysis further provided the evidence that DNase treatment decreased the severe adhesion between scar tissue and the dura mater (Fig. 6b). Moreover, DNase degraded NETs in the scar tissues post laminectomy operation (Fig. 6c). In the H&E staining (Fig. 6d) and Masson staining (Fig. 6e), post-epidural fibrosis in the wound area of the DNase-treated mice was significantly mitigated. In addition, collagen I in the wound tissues was significantly decreased in the DNase-treated mice (Fig. 6f). Collectively, DNase I effectively alleviated scar formation in the mouse model of laminectomy.

## Discussion

Wound repair after a spine operation is a complicated inflammatory process. During the early stage, neutrophils and macrophages infiltrate into the wound area. In the late stage, inflammation recedes, and fibrotic cascades initiate, leading to post-epidural fibrosis. In the present study, we demonstrated that NETs developed in clinical samples from patients post spine surgery and in a mouse laminectomy model in vitro, macrophages ingested NETs, accompanied with increased expression of α-SMA and fibronectin. Fibronectin expression in NETs-treated macrophages was dependent on elastase and NF-κB/Smad3 signaling pathways. DNase, a well-known NETs degrader, alleviated post-epidural fibrosis in the mouse laminectomy model, suggesting that NETs may be a promising candidate for intervention in post-epidural fibrosis.

Emerging evidence has documented that NETs occur in sterile inflammatory diseases^36^. In burn wounds, NETs switched the function of vascular endothelial cells, promoting the formation of intravascular thrombi^37^. Similarly, elevated NETs in diabetic patients were associated with a hypercoagulable state^38^. NETs in diabetic wounds, however, slowed the healing process^16^. The discrepancy regarding NET-mediated regulation of fibrosis may be due to the double-edged effects of NETs^30^. Histones in NETs may damage cells^39^, while low doses of NETs increase cell proliferation and accelerate skin wound healing^40^.

The MMT promotes renal fibrosis, which was reduced in Smad3 knockout mice^21^. As we demonstrated in this study, NETs activated Smad3, and increased the expression of α-SMA and fibronectin in macrophages, suggesting that NETs may induce the transition of macrophages. Once ingested via RAGE, elastase from NETs was transferred into macrophages. We further demonstrated that elastase from NETs or elastase alone potentially promoted the expression of α-SMA and fibronectin in macrophages. An elastase inhibitor attenuated the upregulation of fibronectin in NETs-treated macrophages. As elastase in NETs increased the secretion of α-SMA and favored endometrial fibrogenesis^41^, we hypothesized that elastase from NETs may promote the function transitions of macrophages.

In summary, spine operation caused the elevation of HMGB1, which stimulated the formation of NETs in the wound tissues post spine operation. Macrophages ingested NETs via RAGE, which increased elastase, and activated the NF-κB and Smad3 signaling pathways, leading to the increased expression of α-SMA and fibronectin. DNase disrupted NETs, thereby ameliorating epidural fibrosis in the mouse laminectomy model.

## Methods

### Patients and clinical samples

This study enrolled 14 patients who underwent spinal operation in our department from April 2019 to June 2019. Patients with current or previous systemic autoimmune diseases, or severe infectious diseases were excluded from the study. The research was approved by the ethical committee of the Second Affiliated Hospital of Nanjing Medical University (Nanjing, China) and written informed consent was obtained from all human participants. The postoperative peripheral blood from superficial veins of the upper limb and the postoperative drainage from extracting drainage tube blood were collected. Plasma samples were harvested by centrifugation at 3500 r.p.m. for 5 min and stored at −80 °C for further analysis.

### Mouse model of laminectomy

Specific pathogen-free male C57BL/6 J mice, 8 weeks old, were purchased from the Animal Core Facility of Nanjing Medical University. All mice were acclimated for 1 week before the spine operation. Animal experiments were approved by the Institutional Animal Care and Use Committee of Nanjing Medical University.

The mice were anesthetized by intraperitoneal injection of a mixture of 10 mg/kg xylazine and 200 mg/kg ketamine hydrochloride in 100 μl normal saline. The laminectomy operation was similar as previously described in the rat^42^. Briefly, the L1–L3 vertebral plate was exposed with a midline skin incision, and the dura mater was exposed after removing the spinous process and the L1–L3 vertebral plate with a rongeur. The powder of DNase I was diluted with physiological saline to 1 mg/ml, and the dosage for each mouse was 5 mg/kg (ref. ^34^). The diluted solution was absorbed with a gelatin sponge matching the size of the wound, and it was placed on the wound and then sutured. The control group only used empty gelatin sponge. The incisions were closed by suturing the spinal, fascia, muscle, and skin. The mice were monitored to ensure recovery from anesthesia, and had free access to food and drug.

### NETs induction and purification

Neutrophils were isolated from bone marrow, as previously described^43^. Briefly, the mice were anesthetized and immersed in 70% ethanol. The bone marrow from the femur and tibia was flushed using a sterile syringe filled with phosphate-buffered saline (PBS) and collected into a 50 ml conical tube through a 70 µm cell strainer. Neutrophils from bone marrow cells were negatively selected using a neutrophil magnetic bead isolation kit for sorting granulocytes (130-097-658, Miltenyi Biotec). The purified neutrophils were stimulated with phorbol-12-myristate-13-acetate (PMA, 100 μg/ml, P1585, Sigma) for 4 h at 37 °C to evoke the formation of NETs.

NETs were purified by configuration^44^. In brief, the culture medium was discarded, and the culture plate was gently washed with cold PBS to remove residual PMA and free products from neutrophils. After repeated three times washing, cells undergoing NETs were detached from cell culture plates with thoroughly flushing and harvested into a 10 ml tube. After centrifugation at 450 × *g* for 10 min at 4 °C, the supernatant containing NETs was transferred to a 1.5 ml Eppendorf tube and further centrifuged at 18,000 × *g* for 10 min at 4 °C. The pellets containing NETs with 100 μl residue were collected and stored at −80 °C. OneDrop^TM^ was used to detect the DNA concentration in the NETs.

### Bone marrow-derived macrophage culture

The femurs and tibias were harvested from the mice. Connective tissues and muscles were removed. Both ends of the bones were cut with scissors, and the bone marrow was flushed out with cold PBS with a 31-gauge needle. The cell pellets were collected by centrifugation at 4 °C, and the erythrocytes were lysed with RBC lysis buffer (Thermo Fisher Scientific). The resultant cells were then washed two times with PBS and resuspended in DMEM containing 10% fetal bovine serum (Gibco) supplemented with 100 U/ml penicillin, 0.1 mg/ml streptomycin, and 10 ng/ml recombinant GM-CSF (576308, Biolegend) at a concentration of 2 × 10^6^ cells/ml in a 24-well plate. The bone marrow-derived macrophage (BMDM) culture medium was changed on days 3 and 5. BMDMs were entirely differentiated and ready for use on day 7.

BMDMs (1 × 10^6^) were stimulated with NETs (100 ng/ml) and collected at indicated time points. NETs were pretreated with DNase I (5 μg/ml, DN25, Sigma) to destruct DNA scaffold of NETs and further cultured with BMDMs. To explore the roles of elastase, NETs were pretreated with proteinase K (200 µg/ml, AM2546, Thermofisher) and further cultured with BMDMs. Or, elastase (2 μg/ml, E7885, Sigma) alone was used to culture BMDMs. Alvelestat (20 μg/ml, AZD9668, MCE), BAY-11-7082 (10 μmol, 11-7083, MCE), or SIS3 (10 μmol, HY-13013, MCE) was added into the cell culture medium to block the roles of elastase, NF-κB or Smad-3, respectively.

### Transmission electron microscopy

After incubation with 1000 ng NETs for 4 h, BMDMs (1 × 10^6^) were washed with cold PBS three times, and the cell pellets were fixed in 2.5% glutaraldehyde at 4 °C overnight. The cells were further postfixed with 1% OsO_4_ for 1 h, dehydrated, embedded, and sectioned. The ultrathin slides were observed with a FEI Tecnai G2 Spirit Bio TWIN transmission electron microscope.

### Live cell imaging

Neutrophils (1 × 10^5^/well) were inoculated into a 96-well cell culture plate with macrophages (1 × 10^5^/well), which had been pre-stained with DDAO-SE (2 μg/ml, 468495, Sigma). Four hours after PMA (100 μg/ml, P1585, Sigma) was added to the culture system, the live cell-impermeable dye Sytox Green (1 μM, S11368, Thermo) was added to stain the extracellular DNA. The live phase-gradient comparison images of each region within each well were automatically acquired, using ZEN Blue 2.3 software in the Zeiss Cell Discoverer 7 microscope system.

### Quantification of NETs internalization

To quantify the internalization efficiency of macrophages against NETs, NETs (1000 ng/ml) were cocultured with BMDMs (2 × 10^5^) in a 24-well plate. At various time points, supernatant from each well was harvested, and extracellular DNA was quantified by adding the live cell-impermeable DNA dye Sytox Green (1 μM; S11368, Thermo) and examining the fluorescence intensity with Synergy HTX at an excitation wavelength of 488 nm and an emission wavelength of 523 nm (ref. ^11^).

To explore the roles of RAGE in phagocytosis, the RAGE inhibitor FPS-ZM1 (1 μM, HY-19370, MCE) was added to the cell culture 1 h before adding the NETs. The remaining extracellular DNA from NETs was quantified, as described above.

### Western blot analysis

The cells were lysed on ice in RIPA (P0013B, Beyotime) buffer containing 1 mM PMSF (Beyotime). Equal amounts of protein were separated by 10% SDS–PAGE, and then the proteins were transferred to polyvinylidene fluoride membranes and blocked in 5% bovine serum albumin for 1 h at room temperature. After rinsing in PBST five times, the membranes were incubated with each primary antibody overnight at 4 °C, including HMGB1 (10829-1-AP, Proteintech, 1:1000), citrullinated histone H3 (ab5103, Abcam, 1:1000), fibronectin (15613-1-AP, Proteintech, 1:1000), α- SMA (ab5694, Abcam, 1:200), NF-κB p65 (8242, CST, 1:1000), phospho-NF-κB p65 (3033, CS, 1:1000), Smad3 (9513, CST, 1:1000), phospho-Smad3 (9520, CST, 1:1000), elastase (ab68672, Abcam, 1:1000), and β-actin (4970, CST, 1:1000). After washing with PBST, the membranes were finally incubated with the appropriate secondary antibody for 1 h at room temperature. Protein bands were detected using an ECL high-signal reagent (Thermofisher). We confirmed all blots derive from the same experiment and were processed in parallel.

### RNA isolation and quantitative real-time PCR

Total RNA was obtained from BMDMs with a TRIzol reagent kit (Life Technologies) and reverse-transcribed into cDNA with a reverse transcription kit (Abm, Zhenjiang, China) according to the manufacturer’s instructions. Elastase and β-actin mRNA expression were detected by a StepOnePlus Real-Time PCR System (ABI, USA). The primer sequences used for real-time PCR were designed by referring to PrimerBank (https://pga.mgh.harvard.edu/primerbank). The specific primers for elastase were sense, 5′-AGCAGTCCATTGTGTGAACGG-3′ and anti-sense, 5′-CACAGCCTCCTCGGATGAAG-3′; and for β-actin were sense, 5′-CGTTGACATCCGTAAAGACC-3′ and anti-sense, 5′-AACAGTCCGCCTAGAAGCAC-3′. Relative levels were determined using the 2−ΔΔCt method, and β-actin was used as the internal control. Each sample was run in triplicate, and the results were representative of at least three independent experiments.

### Histological analysis of wound tissues

One month post spine operation, the mice were sacrificed with cervical dislocation. The wound tissues were fixed in 4% paraformaldehyde and embedded in paraffin. For immunofluorescence staining, the wound tissues were cut into 5 μm sections, rinsed, and incubated with the specific primary antibodies for Ly6G (1:100, 13-9668-80, BD Bioscience), F4/80 (1:100, 14-4801-85, BD Bioscience), α-SMA (1:100, 701457, BD Bioscience), MPO (1:100; ab9535, Abcam), and citrullinated histone H3 (Cit-H3 1:50; ab5103, Abcam). All slides were scanned under the same conditions for magnification, exposure time, lamp intensity, and camera gain. The optical density of each positively expressed protein was determined using Image-Pro Plus 6.0 image analysis software.

Alternatively, the wound tissue slides were incubated with H&E, Masson, or primary antibodies against Ly6G or F4/80, followed by horseradish peroxidase-conjugated secondary antibodies. The staining was then performed according to the manual using the ABC staining system (Santa Cruz). 3,3-Diaminobenzidine was used as an indicator. Images of at least three different sections of tissue from each group were acquired by a Zeiss Axio Examiner microscope, and images were analyzed by ImageJ insert IHC Profiler. Image-Pro Plus 6.0 image analysis software was used to determine the area ratio of positively stained collagen.

The surgical sites were reopened through the previous operative incision, and the extent of epidural scar fibrosis was evaluated under double-blind trials according to the Rydell standard^6^. Grade 0, little epidural scar tissue without adherence to the dura mater; grade 1, moderate epidural scar tissue with slight adherence to the dura mater; grade 2, moderate epidural scar tissue with tight adherence to the dura mater and dissected with difficulty without disrupting the dura matter; and grade 3, epidural scar tissue was firmly adherent to the dura mater and could not be dissected.

### Magnetic resonance imaging

MRI was performed 1 month after spine surgery, as described previously^42^. The mice were anesthetized with isoflurane and scanned at the T11-L4 spine. MRI were acquired using a Bruker 7.0 T Micro-MR imaging system and a Multi-Slice Multi-Echo T2-weighted imaging (MSME T2WI) sequence. The time of repetition for MR images was 4391 ms. All parameters (TE, 33.0 ms; layers, 40; thickness, 0.5 mm; and interlayer space, 0) were followed once for a total of 10 min 18 s 120 ms per set. The epidural scar area was measured using ImageJ software^42^.

### Enzyme-linked immunosorbent assay

HMGB1 in the peripheral blood or drainage samples was measured using the HMGB1 direct enzyme-linked immunosorbent assay (ELISA) kit (CK-E11683, Kaerwen, China) according to the manufacturer’s instructions^45^. The absorbance was measured at 450 nm, and the HMGB1 concentration was calculated according to a standard curve.

The spine wound tissue was weighed and homogenized in lysis buffer and centrifuged at 12,000 r.p.m. for 10 min at 4 °C, and the supernatant was taken. The Collagen I ELISA kit (Beijing Longtian Technology Co., Ltd.)^46^ was used to analyze the content of collagen I in the supernatant.

### MPO–DNA complex sandwich ELISA

Soluble NETs, MPO–DNA complex, were assayed as reported before^47^. Briefly, a 96-well plate was coated with MPO antibody (diluted 1/1000, PA5-16672, Thermo Fisher Scientific) overnight at 4 °C, followed by five washing steps and blocking in 1% BSA (A1933, Sigma) for 1 h. Serum samples, diluted 1/10 in 1% BSA, were added into for anti-MPO-coated wells, followed by extensively washing and anti-DNA HRP antibody (diluted 1/100, D5425-3-200, Integratedsci). Following 2 h incubation, the wells were washed five times and 100 µl peroxidase substrate (2023, Invitrogen) was added into each well. The OD at 450 nm was measured after 20 min incubation with having added 2 N sulfuric acid stop solution (C1058, Solarbio) in an ELISA reader (Biotek-synergy).

### Statistics

Data are expressed as the mean ± SEM. Statistical significance was evaluated using GraphPad Prism 7 software (San Diego, CA). Student’s *t* test was performed for two groups. One-way analysis of variance (ANOVA) followed by Tukey’s post hoc test was performed to determine significance among more than two groups. *P* < 0.05 was considered statistically significant.

### Reporting summary

Further information on research design is available in the Nature Research Reporting Summary linked to this article.

## Supplementary information

Supplementary Video 1

Supplementary Information

Reporting Summary

## Data Availability

The data that support the findings of this study are available from the corresponding author upon reasonable request
