# Supplementary material for: Neutrophil extracellular traps promote scar formation in post-epidural fibrosis
Source: NPJ Regen Med. 2020 Oct 30;5:19. doi: 10.1038/s41536-020-00103-1 (PMC7599244; doi:10.1038/s41536-020-00103-1)
Supplement: Supplementary file 2 — Supplementary Information [file 41536_2020_103_MOESM2_ESM.pdf]

Figure 1E

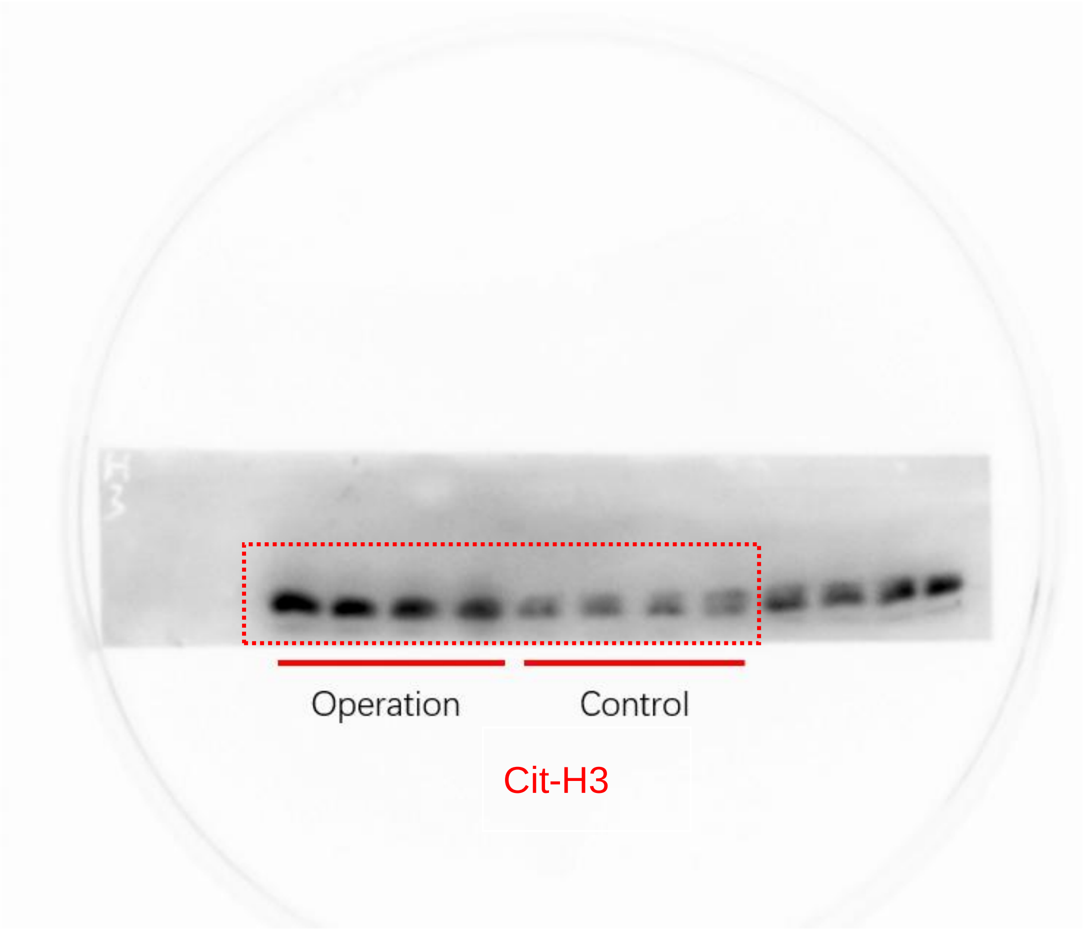

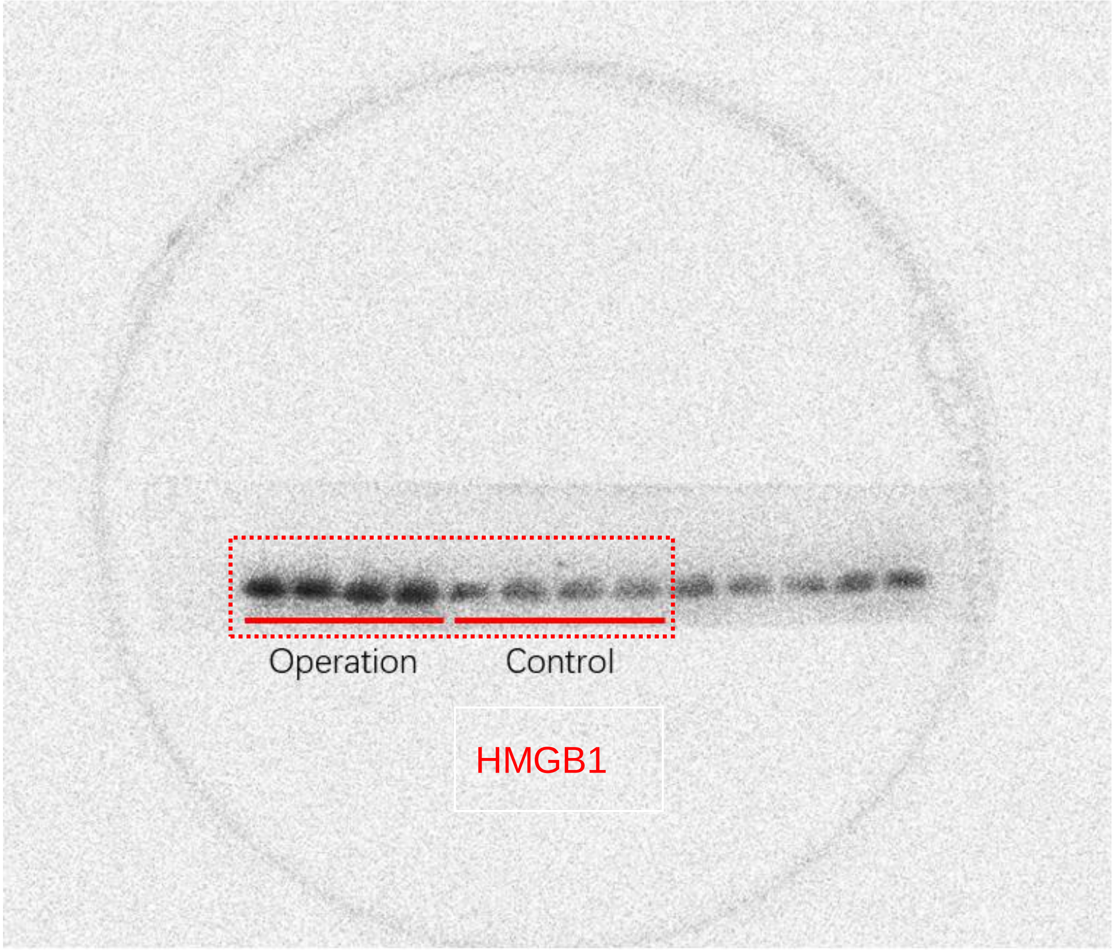
Operation
Control
HMGB1

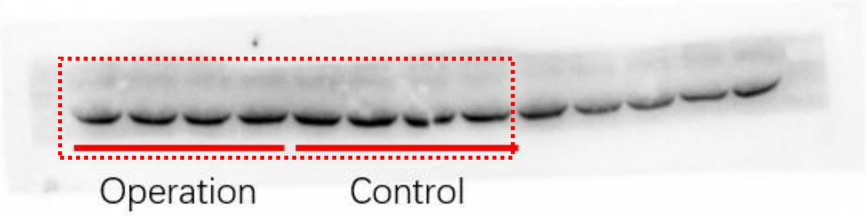


β-actin

Figure 3A

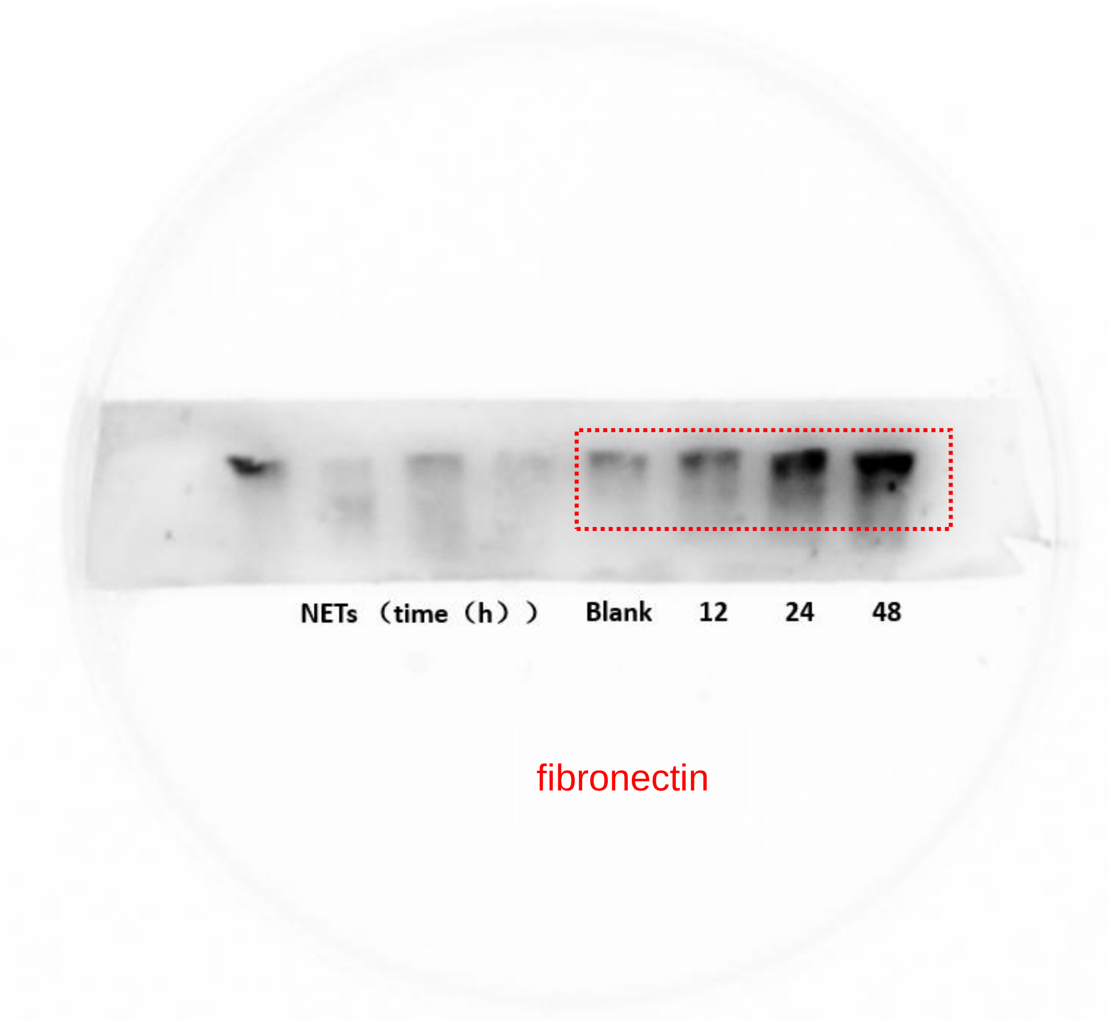

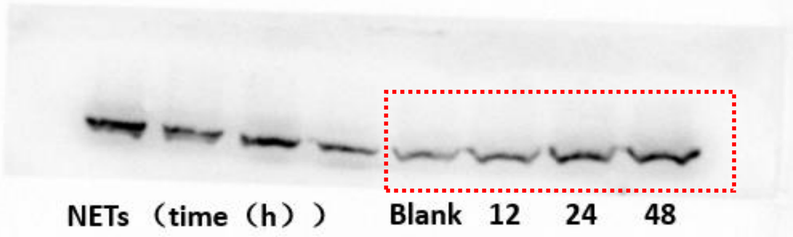


α-SMA

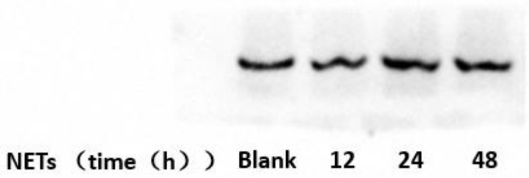


β-actin

Figure 3D

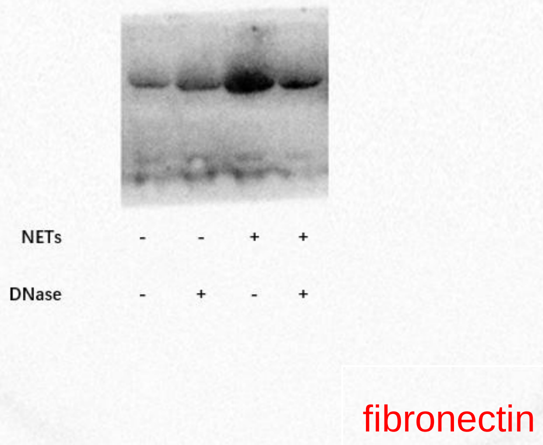


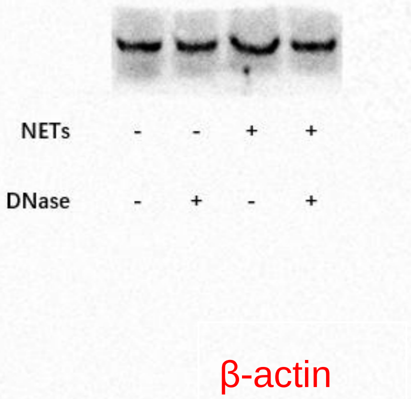

Figure 3E

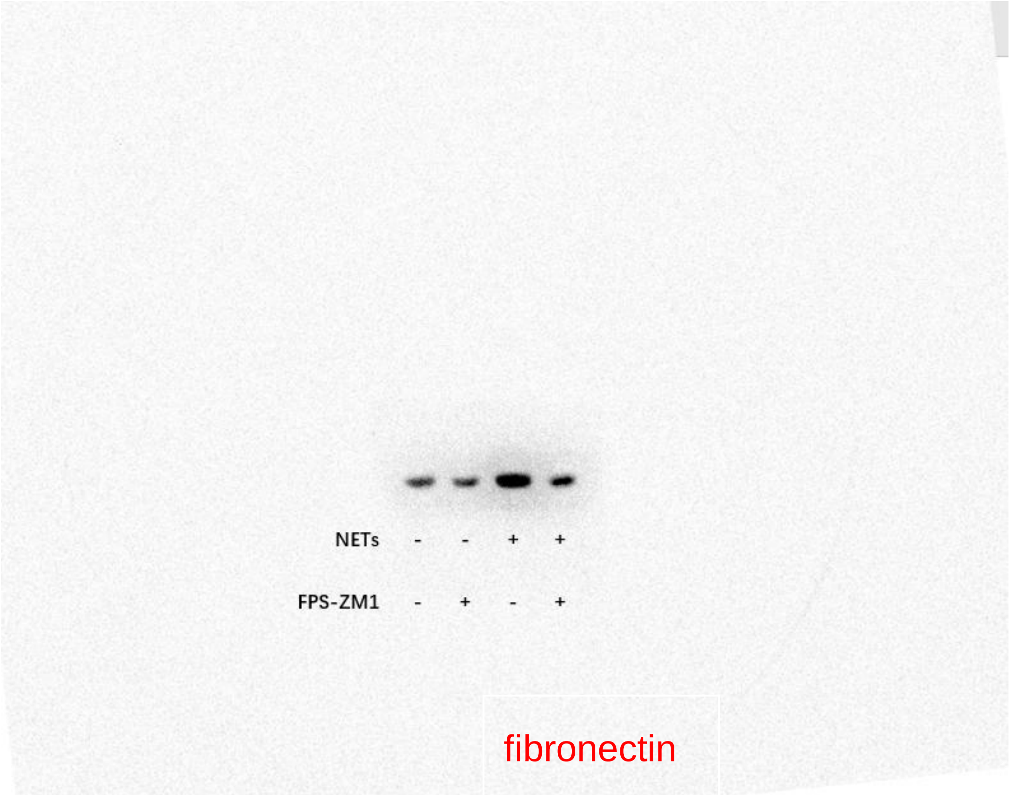


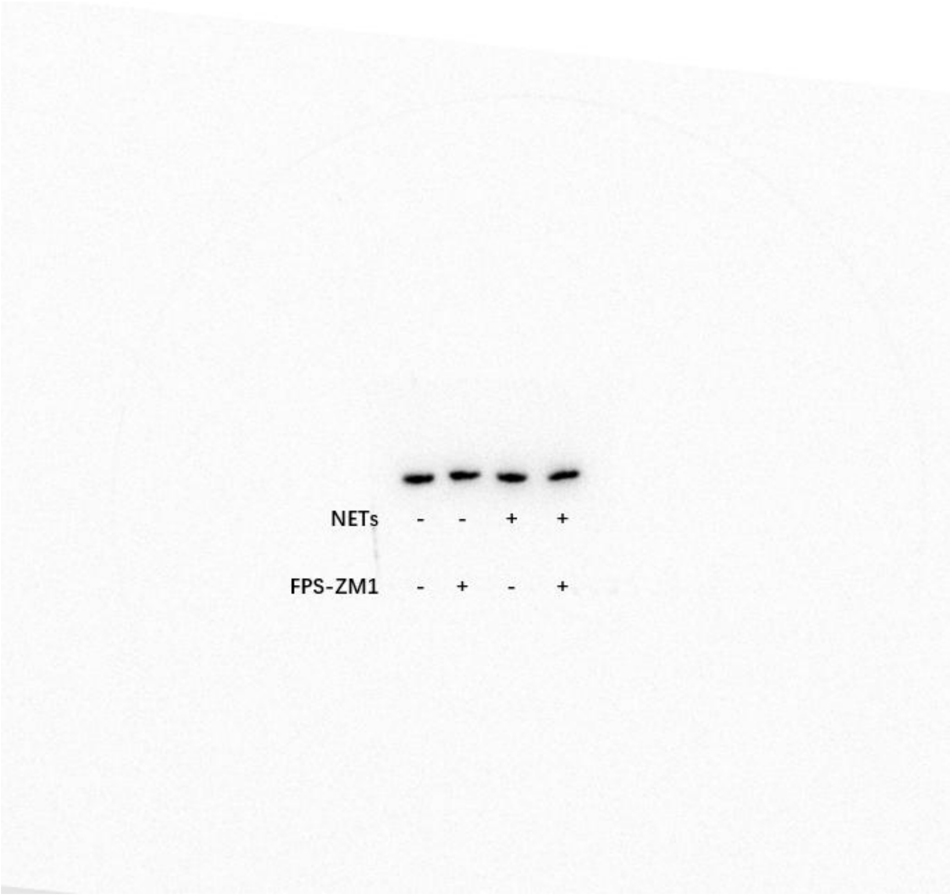

Figure 4A

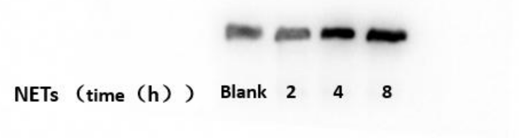


Elastase

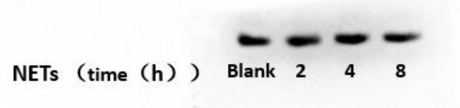


β-actin

Figure 4B

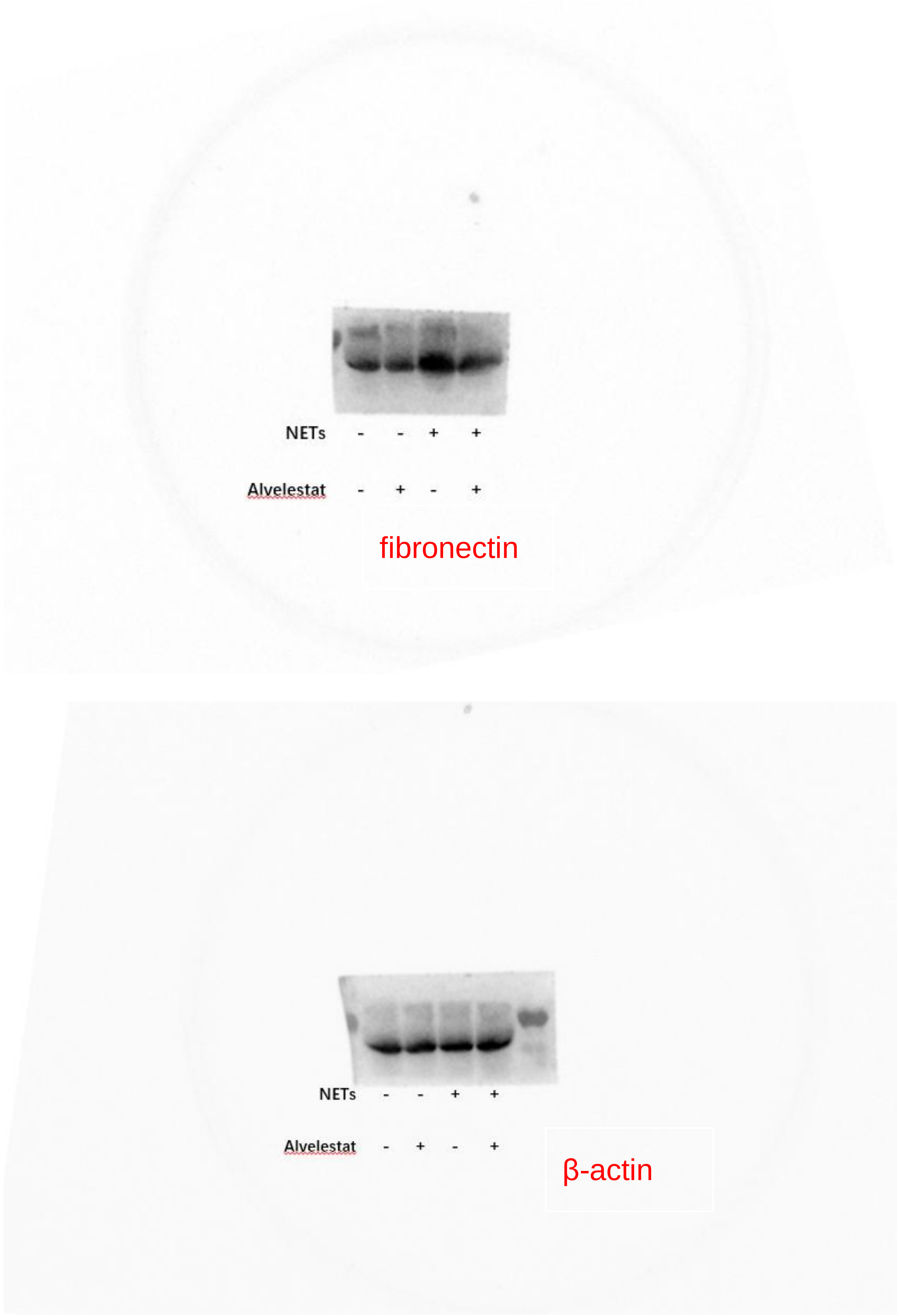

Figure 4C

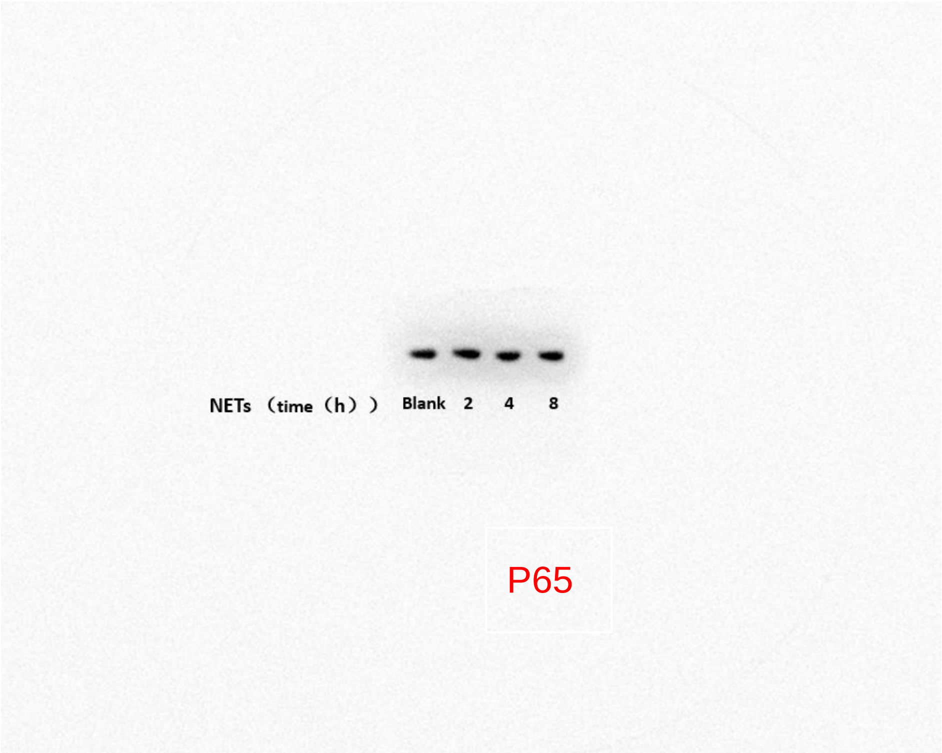


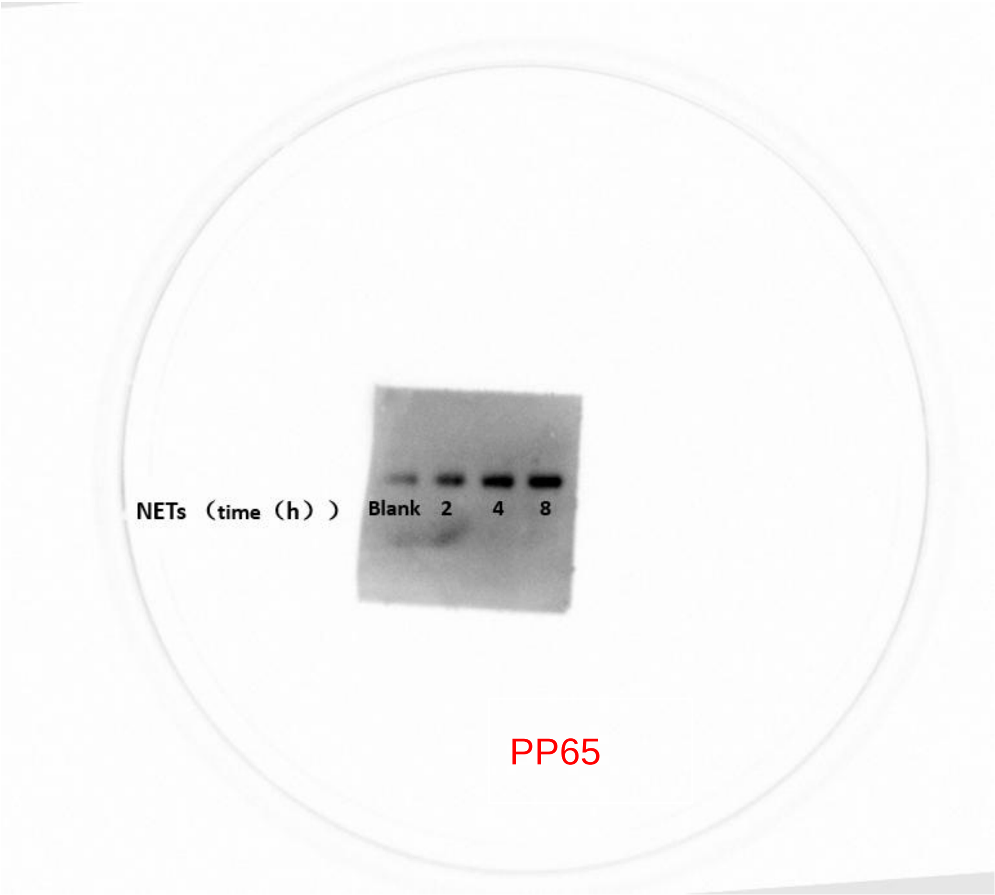

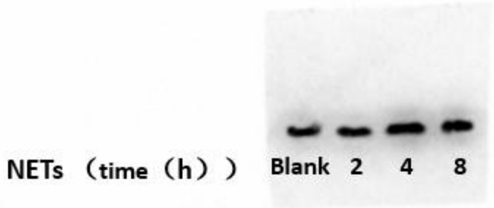


SMAD-3

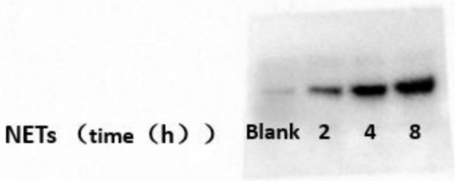


PSMAD-3

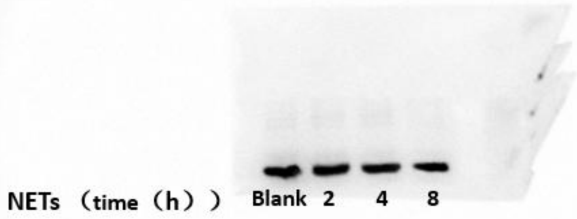


β-actin

Figure 4D

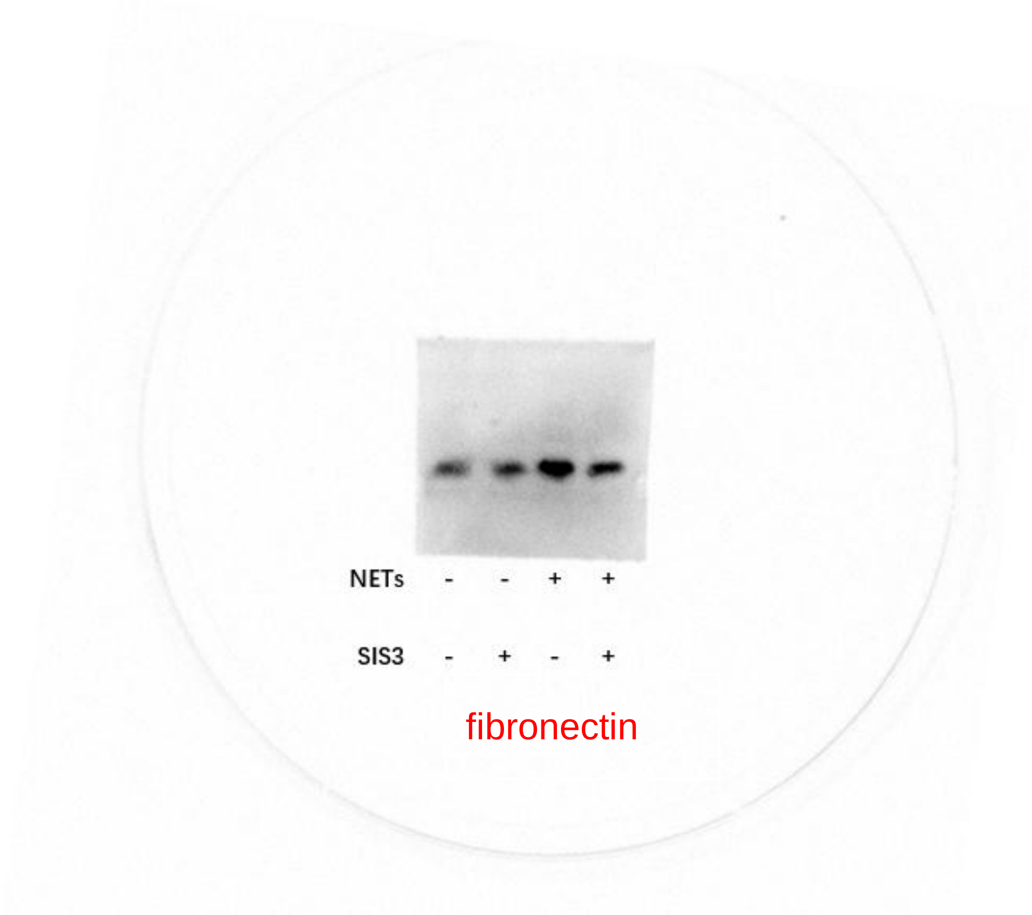


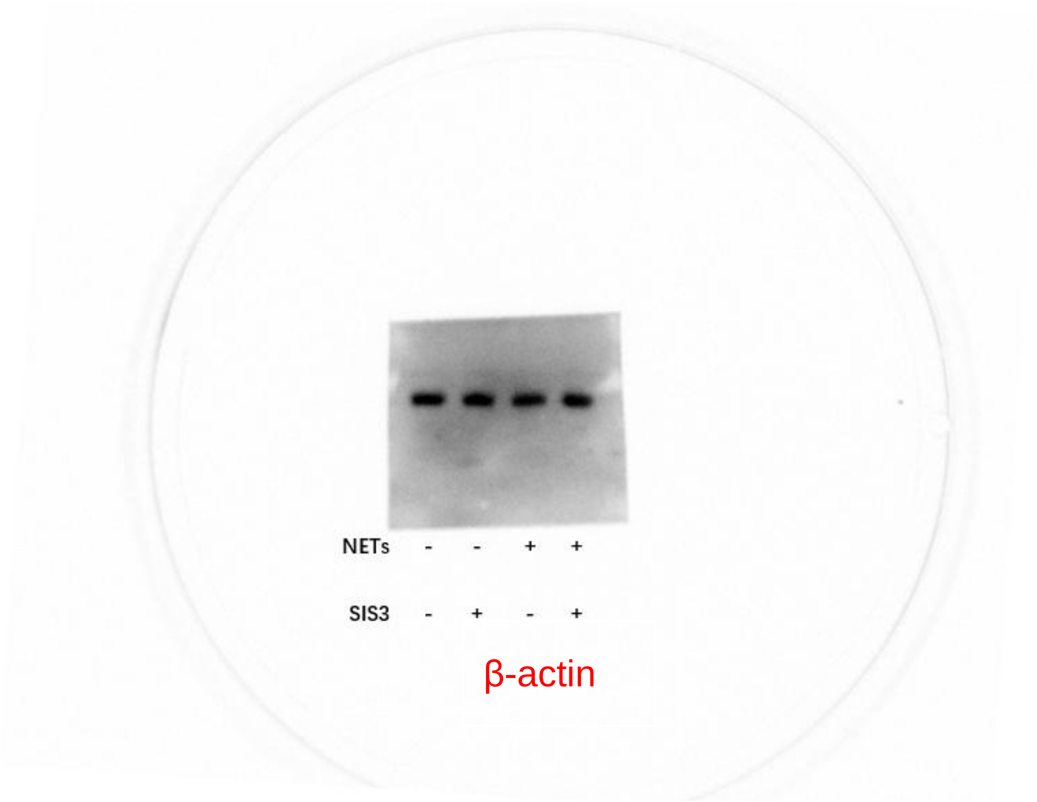

Figure 4E

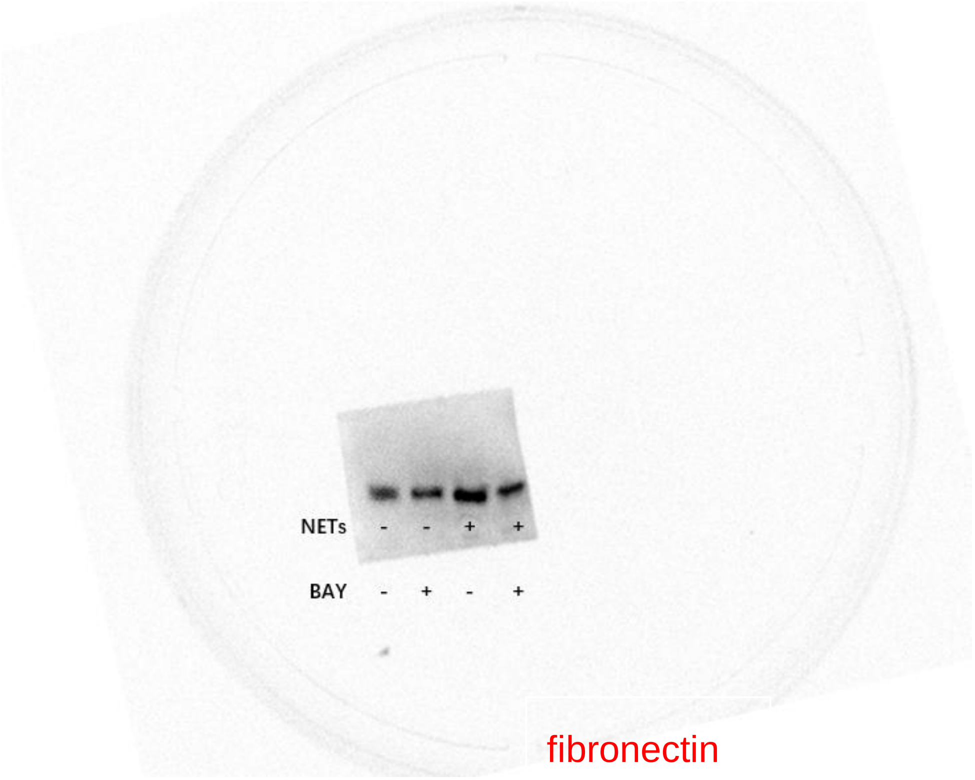


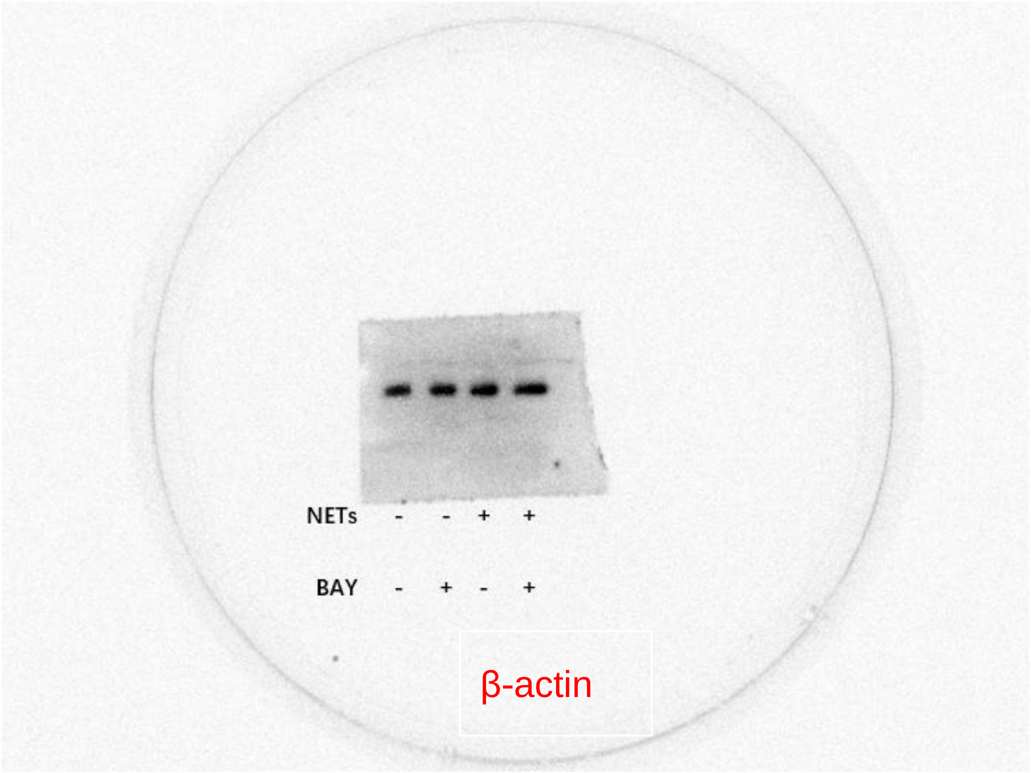

**Supplementary video 1 Live cell imaging of NETs ingested by macrophages.** NETs were labeled with Sytox green (pseudo-green); macrophages were labeled with DDAO-SE (pseudo-red); live cell imaging showed that NETs became yellow if ingested into macrophages.
