# Supplementary material for: Neutrophil extracellular traps promote scar formation in post-epidural fibrosis
Source: NPJ Regen Med. 2020 Oct 30;5:19. doi: 10.1038/s41536-020-00103-1 (PMC7599244; doi:10.1038/s41536-020-00103-1)
Supplement: Supplementary file 3 — Reporting Summary [file 41536_2020_103_MOESM3_ESM.pdf]

nature research

Corresponding author(s): NPJREGENMED-00343R2

Last updated by author(s): Sep 10, 2020

Reporting Summary

Nature Research wishes to improve the reproducibility of the work that we publish. This form provides structure for consistency and transparency in reporting. For further information on Nature Research policies, see our Editorial Policies and the Editorial Policy Checklist.

Statistics

For all statistical analyses, confirm that the following items are present in the figure legend, table legend, main text, or Methods section.

n/a	Confirmed	
☐	☒	The exact sample size (n) for each experimental group/condition, given as a discrete number and unit of measurement
☐	☒	A statement on whether measurements were taken from distinct samples or whether the same sample was measured repeatedly
☐	☒	The statistical test(s) used AND whether they are one- or two-sided<br>*Only common tests should be described solely by name; describe more complex techniques in the Methods section.*
☐	☒	A description of all covariates tested
☐	☒	A description of any assumptions or corrections, such as tests of normality and adjustment for multiple comparisons
☐	☒	A full description of the statistical parameters including central tendency (e.g. means) or other basic estimates (e.g. regression coefficient) AND variation (e.g. standard deviation) or associated estimates of uncertainty (e.g. confidence intervals)
☒	☐	For null hypothesis testing, the test statistic (e.g. F, t, r) with confidence intervals, effect sizes, degrees of freedom and P value noted<br>*Give P values as exact values whenever suitable.*
☒	☐	For Bayesian analysis, information on the choice of priors and Markov chain Monte Carlo settings
☒	☐	For hierarchical and complex designs, identification of the appropriate level for tests and full reporting of outcomes
☒	☐	Estimates of effect sizes (e.g. Cohen's d, Pearson's r), indicating how they were calculated

Our web collection on statistics for biologists contains articles on many of the points above.

Software and code

Policy information about availability of computer code

Data collection	No software was used.
Data analysis	No software was used.

For manuscripts utilizing custom algorithms or software that are central to the research but not yet described in published literature, software must be made available to editors and reviewers. We strongly encourage code deposition in a community repository (e.g. GitHub). See the Nature Research guidelines for submitting code & software for further information.

Data

Policy information about availability of data

All manuscripts must include a data availability statement. This statement should provide the following information, where applicable:

- Accession codes, unique identifiers, or web links for publicly available datasets
- A list of figures that have associated raw data
- A description of any restrictions on data availability

The data that support the findings of this study are available from the corresponding author upon reasonable request.

Field-specific reporting

Please select the one below that is the best fit for your research. If you are not sure, read the appropriate sections before making your selection.

☒ Life sciences ☐ Behavioural & social sciences ☐ Ecological, evolutionary & environmental sciences

For a reference copy of the document with all sections, see nature.com/documents/nr-reporting-summary-flat.pdf

Life sciences study design

All studies must disclose on these points even when the disclosure is negative.

Sample size	More than 5 biological samples were used in different groups.
Data exclusions	No data was excluded from the study.
Replication	The results were successfully repeated.
Randomization	The mice were randomly allocated into control group, operation group, or operation group with DNase treatment.
Blinding	We confirmed the investigators were blinded to group allocation during data collection and/or analysis.

Reporting for specific materials, systems and methods

We require information from authors about some types of materials, experimental systems and methods used in many studies. Here, indicate whether each material, system or method listed is relevant to your study. If you are not sure if a list item applies to your research, read the appropriate section before selecting a response.

Materials & experimental systems

n/a	Involved in the study	
☐	☒	Antibodies
☐	☒	Eukaryotic cell lines
☒	☐	Palaeontology and archaeology
☐	☒	Animals and other organisms
☐	☒	Human research participants
☐	☐	Clinical data
☐	☐	Dual use research of concern

Methods

n/a	Involved in the study	
☒	☐	ChIP-seq
☒	☐	Flow cytometry
☐	☒	MRI-based neuroimaging

Antibodies

Antibodies used	The antibodies were listed in Table 1.
Validation	The antibodies were validated before the experiments.

Eukaryotic cell lines

Policy information about cell lines

Cell line source(s)	No cell line was used.
Authentication	No cell line was used.
Mycoplasma contamination	We confirmed our primary cell culture was negative for contamination.
Commonly misidentified lines (See ICLAC register)	No cell line was used.

Animals and other organisms

Policy information about studies involving animals; ARRIVE guidelines recommended for reporting animal research

Laboratory animals	male C57BL/6J mice

Wild animals	No wild animals were involved in the study.
Field-collected samples	No filed-collected samples were involved in the study.
Ethics oversight	Animal experiments were approved by the Institutional Animal Care and Use Committee (IACUC) of Nanjing Medical University.

Note that full information on the approval of the study protocol must also be provided in the manuscript.

Human research participants

Policy information about studies involving human research participants

Population characteristics	This study enrolled 14 patients who underwent spinal operation in our department from April 2019 to June 2019.
Recruitment	Patients with current or previous systemic autoimmune diseases or severe infectious diseases were excluded from the study.
Ethics oversight	The research was approved by the ethical committee of the Second Affiliated Hospital of Nanjing Medical University (Nanjing, China)

Note that full information on the approval of the study protocol must also be provided in the manuscript.

Clinical data

Policy information about clinical studies

All manuscripts should comply with the ICMJE guidelines for publication of clinical research and a completed CONSORT checklist must be included with all submissions.

Clinical trial registration	Clinical trial is not involved with the present study.
Study protocol	Clinical trial is not involved with the present study.
Data collection	Clinical trial is not involved with the present study.
Outcomes	Clinical trial is not involved with the present study.

Dual use research of concern

Policy information about dual use research of concern

Hazards

Could the accidental, deliberate or reckless misuse of agents or technologies generated in the work, or the application of information presented in the manuscript, pose a threat to:

No	Yes	
☒	☐	Public health
☒	☐	National security
☒	☐	Crops and/or livestock
☒	☐	Ecosystems
☒	☐	Any other significant area

Experiments of concern

Does the work involve any of these experiments of concern:

No	Yes	
☒	☐	Demonstrate how to render a vaccine ineffective
☒	☐	Confer resistance to therapeutically useful antibiotics or antiviral agents
☒	☐	Enhance the virulence of a pathogen or render a nonpathogen virulent
☒	☐	Increase transmissibility of a pathogen
☒	☐	Alter the host range of a pathogen
☒	☐	Enable evasion of diagnostic/detection modalities
☒	☐	Enable the weaponization of a biological agent or toxin
☒	☐	Any other potentially harmful combination of experiments and agents

Magnetic resonance imaging

Experimental design

Design type	indicate design
Design specifications	The experiment was divided into three groups (operation, sham operation,operation+ DNase), each group of 6 mice, the mice underwent spinal surgery at 8 weeks, and continued to be fed for 4 weeks after the operation, and performed MRI scan.
Behavioral performance measures	none

Acquisition

Imaging type(s)	3D MRI （Biospec 7T/20 USR）
Field strength	none
Sequence & imaging parameters	TE, 33.0 ms; layers, 40; thickness, 0.5 mm; and interlayer space, 0
Area of acquisition	Scanning the whole torso of mice

Diffusion MRI ☐ Used ☒ Not used

Preprocessing

Preprocessing software	Biospec 7T/20 USR
Normalization	The sham operated group (Control) without epidural scars was found no high-signal scars on the epidural, so it was regarded as the standard, and the area of the scars expressed as high-signal shadows in the Operation group and DNase + Operation group was measured with Image-Pro Plus 6.0.
Normalization template	Use the sham operation group (Control) without epidural scar as a normalization template
Noise and artifact removal	Respiration was monitored using a pressure sensor connected to an air-filled balloon positioned under the abdomen,heart rate and respiratory rate were continuously monitored throughout themonitoring system (Small Animal Instruments Inc., Stony Brook, NY, USA)
Volume censoring	Measuring scar area with Image-Pro Plus 6.0.

Statistical modeling & inference

Model type and settings	Select the cross-section of the middle area of the surgical wound to measure the epidural scar area
Effect(s) tested	The weight and volume of the same batch of mice are basically the same directly using non-parametric t test

Specify type of analysis: ☒ Whole brain ☐ ROI-based ☐ Both

Statistic type for inference (See Eklund et al. 2016)	non-parametric t test
Correction	none

Models & analysis

n/a	Involved in the study
☒	☐ Functional and/or effective connectivity
☒	☐ Graph analysis
☒	☐ Multivariate modeling or predictive analysis
